# Hybrid Vision Transformer–CNN Architecture with Optimized Feature Selection for Skin Cancer Classification

**DOI:** 10.3390/diagnostics16152351

**Published:** 2026-07-27

**Authors:** Abrar Almjally, Munazza Aziz, Shaheryar Najam, Alaa Menshawi, Meteb Altaf, Abdullah Fawaz Aljulayfi, Ahmad Jalal

**Affiliations:** 1 Information Technology Department, College of Computer and Information Sciences, Imam Mohammad Ibn Saud Islamic University (IMSIU), Riyadh 13318, Saudi Arabia; aamjally@imamu.edu.sa; 2 Department of Biomedical Engineering, Riphah International University, I-14, Islamabad 44000, Pakistan; azizmunazza068@gmail.com; 3 Department of Electrical Engineering, Bahria University, H-11, Islamabad 44000, Pakistan; shaheryarnajam.h11@bahria.edu.pk; 4 Information Systems Department, College of Computer and Information Sciences, Imam Mohammad Ibn Saud Islamic University (IMSIU), Riyadh 13318, Saudi Arabia; amenshawi@imamu.edu.sa; 5 Disability Research Institute, Health Sector, King Abdulaziz City for Science and Technology Riyadh, Riyadh 11442, Saudi Arabia; 6 Department of Software Engineering, College of Computer Engineering and Sciences, Prince Sattam Bin Abdulaziz University, Al-Kharj 16273, Saudi Arabia; a.aljulayfi@psau.edu.sa; 7 Department of Computer Science, Air University, E-9, Islamabad 44000, Pakistan; ahmadjalal@mail.au.edu.pk; 8 Department of Computer Science and Engineering, College of Informatics, Korea University, Seoul 02841, Republic of Korea

**Keywords:** melanoma detection, skin lesion classification, deep learning, dermoscopic image analysis, feature extraction, attention mechanism, medical image processing

## Abstract

**Background/Objectives:** Melanoma is a life-threatening skin cancer characterized by aggressive progression and high metastatic potential, making early diagnosis essential for improving patient survival and treatment outcomes. However, accurate automated skin lesion classification remains challenging due to variations in lesion appearance, illumination, image quality, and the presence of artifacts. This study proposes a unified framework for robust multi-class skin cancer classification by integrating preprocessing, lesion segmentation, feature extraction, optimization, and classification within a single end-to-end architecture. **Methods:** The proposed framework employs an iterative hair artifact removal strategy based on the fusion of Frangi vesselness filtering, Gabor texture filtering, morphological refinement, and Telea inpainting to preserve lesion integrity. A novel dermoscopic lesion segmentation network (CutisNet) is introduced to accurately delineate lesion boundaries. Hybrid representation learning combines deep features extracted using MobileNetV2 with uniquely selected handcrafted descriptors to capture complementary texture, structural, and contextual information. Gray Wolf Optimization is utilized for feature fusion and refinement, while a hybrid GNN–CNN classifier performs robust multi-class skin lesion classification. **Results:** Extensive experiments conducted on multiple benchmark dermoscopic datasets demonstrate the effectiveness and generalization capability of the proposed framework. The proposed model consistently outperformed existing state-of-the-art methods, achieving a maximum classification accuracy of 96.7% on the PH^2^ dataset while maintaining competitive performance across other benchmark datasets. **Conclusions:** The proposed framework effectively integrates novel preprocessing, segmentation, hybrid feature representation, feature optimization, and classification strategies to improve the robustness and accuracy of automated skin cancer classification. These results demonstrate its potential to support reliable computer-aided diagnosis and assist clinicians in the early detection of skin cancer.

## 1. Introduction

Skin cancer detection is a focal point in modern medical research due to the growing increase in dermatological disorders, especially malignant skin lesions. Melanoma is the most serious type of skin cancer because it grows rapidly and can spread to other parts of the body. An upsurge in incidence over the last decade has been reported in recent epidemiology studies, which are associated with factors including extended exposure to the ultraviolet rays of the sun, environmental changes, and aging populations [1]. Early diagnosis is crucial as the prognosis and treatment options are limited if the lesions are diagnosed late, whereas prognosis is improved and treatment options are broader in the early stages of diagnosis [2].

The current diagnostic methods, such as visual inspection and dermoscopic assessment, are highly dependent on the clinician’s experience and image interpretation. This frequently leads to intra- and inter-observer variability, particularly when diagnosing early-stage lesions with subtle visual features [2]. Artificial Intelligence (AI)-based computer-aided diagnostic systems help reduce this subjectivity by providing objective and reproducible image analysis that is independent of the clinician’s level of experience. Moreover, deep learning, particularly hybrid feature fusion frameworks that integrate deep and handcrafted representations, can capture subtle texture, shape, and morphological variations that may not be readily distinguishable through visual assessment alone. Although AI systems remain challenged by difficult cases and should not replace clinical expertise, they can serve as valuable decision-support tools that improve diagnostic consistency and assist clinicians in skin cancer screening.

In addition to conventional dermoscopic imaging, emerging technologies such as Raster-Scan Optoacoustic Mesoscopy (RSOM), photoacoustic tomography, and Raman spectroscopy have shown promise for skin cancer diagnosis by providing vascular, structural, and biochemical tissue information [3]. However, these modalities require specialized imaging equipment and clinical infrastructure, limiting their widespread adoption. In contrast, AI-based analysis of conventional RGB dermoscopic and clinical images offers a more practical, accessible, and cost-effective solution for routine computer-aided skin cancer screening and diagnosis.

Large-scale annotated datasets have played a vital role in recent times, in the field of automated skin cancer detection in this context. There is a vast amount of publicly available material, like the ISIC archive, which contains a wide range of dermoscopic images for a large number of different categories of lesions, which have greatly helped research by providing a wide variety of images to use for benchmarking and comparative analysis [4]. Annotated high-quality images with well-defined classes of lesions have been provided by other datasets (PH^2^, HAM10000), which allow for both segmentation and classification [4]. Moreover, datasets such as PAD UFES 20 also contain clinical metadata, enabling multimodal analysis and enhancing a model’s generalization. However, these datasets are especially prone to class imbalance and have limited representation of rare malignancies and variations in imaging conditions, making the development of robust models difficult.

Initial computational methods relied on manually engineered features from these datasets, such as color, texture, and shape descriptors, as well as classic machine learning classifiers. Those methods offered a set of rules with a comprehensible interpretation, but were these constrained by the human work of feature engineering and by their sensitivity to changes in illumination and image quality [4]. Deep learning, especially deep convolutional neural networks, has revolutionized the field of skin cancer detection, allowing for automatic feature learning directly from image data. These models have been shown to have high accuracy and, in some instances, performance similar to dermatologists [5]. However, they are highly dependent on the availability of extensive and diverse datasets, and are prone to overfitting and bias if they are trained on small or imbalanced sets of data.

In this work, a unified skin lesion analysis framework is proposed that integrates preprocessing, deep learning, segmentation, feature extraction, optimization, and classification. Dermoscopic RGB images are first preprocessed, followed by iterative Frangi- and Gabor-based hair removal and Telea inpainting to enhance lesion visibility. The refined images are then used for deep feature extraction using a ViT-based DINO and MobileNetV2 to capture complementary global and local representations. In parallel, a novel segmentation model, CutisNet, generates precise RGB lesion masks, which are further used to extract handcrafted features, including SIFT keypoints, superpixel centroid and contour points, texture descriptors using Tamura features, GLRLM, and Gabor filters, along with structural features based on symmetry analysis, Hu moments, and ellipse fitting. Finally, all deep, texture, keypoint, and structural features are fused, optimized using Gray Wolf Optimization, and then classified using a hybrid GNN-CNN architecture for final skin cancer classification.

The key contributions of the proposed model are summarized as follows:

1. A unified hybrid framework for automated skin cancer classification is proposed, integrating preprocessing, novel lesion segmentation, hybrid deep and handcrafted feature representation, feature optimization, and hybrid classification within a single end-to-end architecture. Unlike conventional pipelines that treat these stages independently, the proposed framework enables complementary interaction among all modules, resulting in improved robustness, generalization, and multi-class classification performance.
2. A novel dermoscopic lesion segmentation network, termed CutisNet, is introduced to accurately delineate lesion boundaries through hierarchical feature encoding, multi-scale contextual aggregation, and coarse-to-fine decoding. By effectively preserving lesion morphology and boundary details under challenging imaging conditions, CutisNet achieves superior segmentation performance compared with established architectures, including U-Net, U-Net++, and DeepLabV3+.
3. A novel iterative hair artifact removal strategy is developed by integrating Frangi vesselness filtering, Gabor texture filtering, morphological refinement, and Telea inpainting. The complementary strengths of vessel enhancement and texture analysis enable effective suppression of both linear and curved hair artifacts while preserving lesion integrity and minimizing the loss of diagnostically relevant structures.
4. A unique hybrid representation learning strategy is proposed by integrating deep features extracted from MobileNetV2 and a self-supervised ViT-based DINO with uniquely selected handcrafted descriptors, including textural, geometric, symmetric, and keypoint-based features. The complementary fusion of local texture information, global contextual dependencies, structural characteristics, and clinically relevant handcrafted descriptors produces a more discriminative and robust feature representation than any individual feature extraction approach.
5. A novel hybrid GNN–CNN classification framework is proposed to jointly exploit graph-based relational learning and convolutional feature learning for multi-class skin lesion classification. The CNN effectively captures discriminative local lesion patterns, while the GNN models structural relationships among the optimized feature representations, enabling improved feature interaction, enhanced classifier generalization, and more accurate discrimination of visually similar skin lesion categories. Extensive experiments, comparative evaluations, and ablation studies on benchmark datasets, including International Skin Imaging Collaboration—ISIC, PAD-UFES-20, Derm7pt, BCN20000, and PH2, demonstrate the superior effectiveness, generalization capability, and contribution of the proposed framework over existing state-of-the-art methods.

## 2. Literature Review

Skin cancer has become one of the fastest growing cancers globally, with melanoma responsible for a large proportion of skin cancer related mortality due to its aggressive and metastatic nature. Epidemiological studies have reported a steady rise in incidence, particularly in populations with high ultraviolet exposure and aging demographics, emphasizing the increasing disease burden [1]. The prevalence of late-stage diagnoses further underscore the necessity of early detection, as survival rates decrease significantly with disease progression [2]. Consequently, automated diagnostic systems are being increasingly developed to assist clinicians, reduce subjectivity, and enable scalable screening.

Conventional diagnostic methods, such as dermoscopy and visual inspection, are highly dependent on clinician expertise, leading to variability in diagnosis. Early computational approaches addressed this by extracting handcrafted features including color distribution, asymmetry, border irregularity, texture, and shape descriptors, followed by classification using machine learning algorithms, such as support vector machines, k-nearest neighbors, and random forests. Although these methods provided interpretability and a structured framework, they were limited by low feature discriminative power, sensitivity to imaging conditions, and reliance on expert-designed features [6].

Deep learning, particularly convolutional neural networks, significantly improved skin cancer detection by enabling automatic hierarchical feature learning from raw images. These models demonstrated high accuracy by capturing complex spatial and contextual patterns and have achieved performance comparable to dermatologists in certain tasks [5]. Architectures such as ResNet and DenseNet further enhanced performance through improved gradient flow and feature reuse [7]. However, their dependence on large, annotated datasets and susceptibility to overfitting in limited data scenarios remain key limitations.

Segmentation based methods have been employed to improve lesion localization prior to classification, reducing background noise and enhancing feature extraction. Encoder–decoder architectures, such as U Net and fully convolutional networks, have shown strong performance in delineating lesion boundaries under challenging conditions [8]. Despite their effectiveness, segmentation errors due to low contrast, artifacts, or irregular lesion shapes can propagate and degrade classification accuracy. Hybrid and ensemble approaches have been proposed to improve robustness by combining multiple models or integrating handcrafted and deep features. Attention mechanisms and multi-scale feature fusion further enhance the focus on clinically relevant regions and improve feature representation across scales [9]. While these methods achieve higher accuracies, they introduce increased computational complexity and reduced interpretability, limiting their practicality in real-world clinical settings.

Transformer-based architectures have recently gained attention due to their ability to model global dependencies and contextual relationships within images. Vision Transformers and hybrid CNN transformer models show promising performance in capturing global lesion characteristics [10]. However, their high computational requirements and reliance on large-scale annotated datasets restrict their widespread application in medical imaging. Transfer learning has been widely adopted to mitigate data scarcity by leveraging pretrained models, improving convergence speed and classification performance [11]. Nevertheless, domain mismatch between natural and medical images can limit feature relevance, requiring careful fine tuning for reliable results.

Despite these advancements, several challenges persist. Dataset imbalance, where malignant cases are underrepresented, leads to biased predictions and reduced sensitivity [6]. Variations in imaging conditions, acquisition devices, and annotation standards hinder generalization across clinical settings [12]. Additionally, limited interpretability of deep learning models raises concerns regarding trust and clinical adoption [13]. In summary, while deep learning and advanced hybrid models have substantially improved skin cancer detection, the increasing disease burden highlights the need for robust, generalizable, and interpretable systems that can effectively operate in real-world clinical environments.

## 3. Materials and Methods

**Figure 1:**
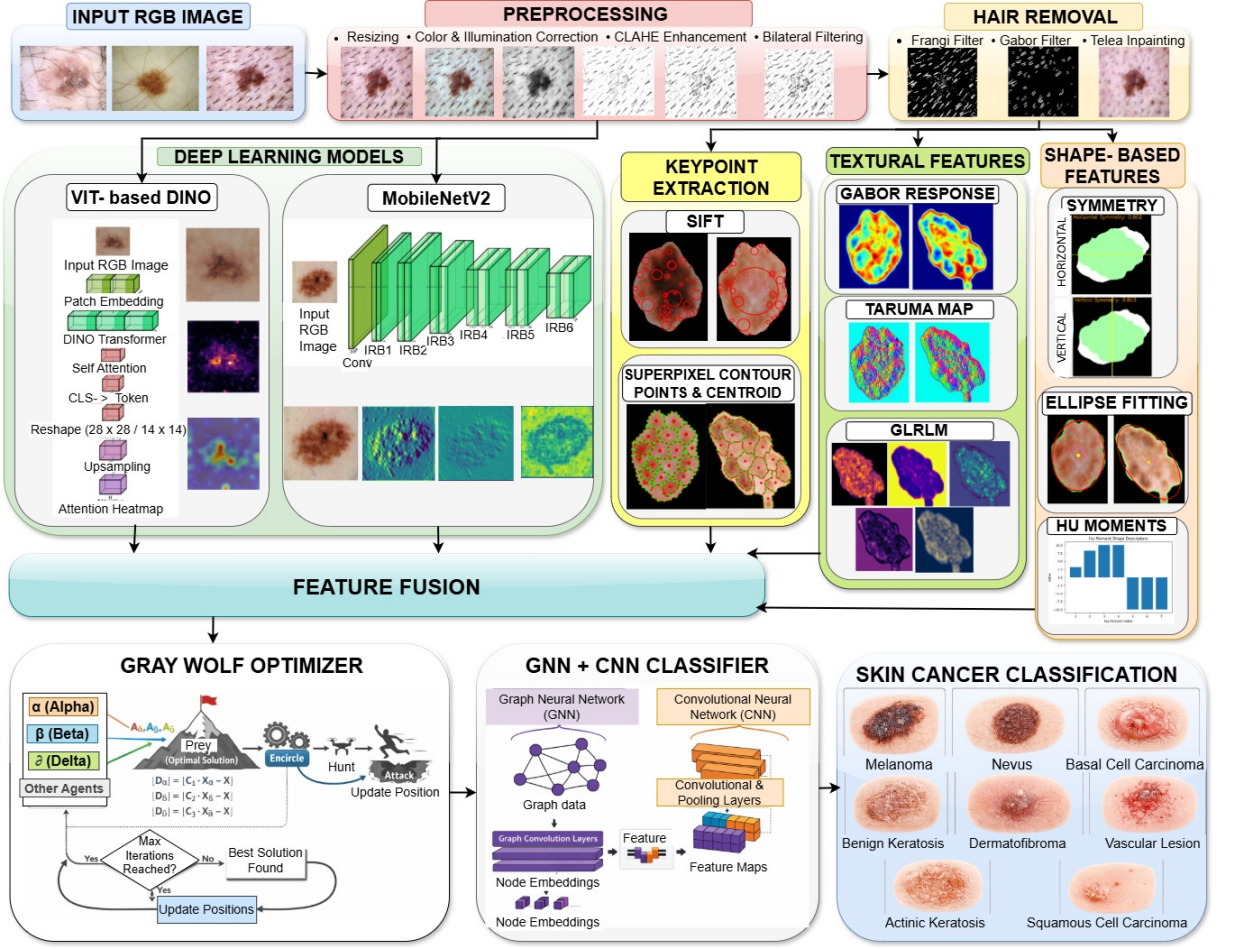
Architecture of the proposed skin cancer classification model.

The proposed methodology presents a unified framework for skin lesion analysis by integrating preprocessing, segmentation, feature extraction, optimization, and classification into a single pipeline. It leverages both deep and handcrafted features to capture comprehensive lesion characteristics. The overall architecture of the proposed system is illustrated in Figure 1.

### 3.1. Image Preprocessing and Enhancement

**Figure 2:**
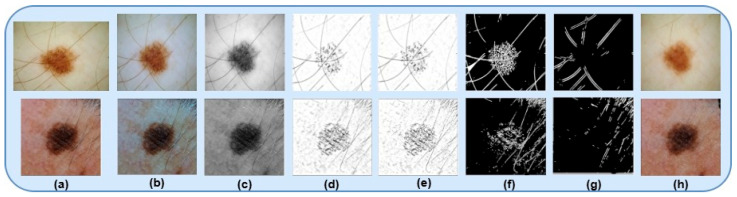
Preprocessing and hair artifact removal of ISIC (top row) and PAD-UFES-20 (bottom row): (**a**) original image; (**b**) resized and color-corrected image; (**c**) grayscale image (**d**) illumination-corrected and enhanced image; (**e**) smoothed image; (**f**) Frangi mask; (**g**) Gabor mask; and (**h**) hair-removed image.

A multi-stage preprocessing pipeline was employed to enhance dermoscopic image quality and consistency, including color normalization, illumination correction, contrast enhancement, and noise reduction. Initially, gray world color normalization corrected color imbalance. We let I(x,y,c) denote the RGB image, displayed in Figure 2a, where c∈{R,G,B}. The mean intensity of each channel is $\mu_{c}=\frac{1}{N}\sum_{x,y}I(x,y,c)$, and the global gray average is $\mu_{\mathrm{gray}}$. Each channel is scaled to enforce color constancy, as expressed by Equation (1), producing Figure 2b.

(1) $$\mu_{\mathrm{gray}}=\frac{\mu_{R}+\mu_{G}+\mu_{B}}{3},{\;\;\;\;\;I}_{\mathrm{norm}}(x,y,c)=I(x,y,c)\cdot \frac{\mu_{\mathrm{gray}}}{\mu_{c}}$$

The normalized image was converted to grayscale $I_{g}(x,y)$, as shown in Figure 2c, followed by illumination correction to suppress uneven lighting effects. A smoothed illumination estimate $L\left(x,y\right)$ was obtained using Gaussian filtering as $L(x,y)=G_{\sigma }(x,y)*I_{g}(x,y),$ and the corrected image was computed using homomorphic normalization with Equation (2)

(2) $$I_{\mathrm{corr}}(x,y)=\frac{I_{g}(x,y)}{L(x,y)}\cdot 255$$

Contrast was enhanced using CLAHE, yielding Figure 2d. Finally, bilateral filtering performed edge-preserving smoothing. The filtered image $I_{f}(x,y)$, as shown in Figure 2e, is defined as Equation (3).

(3) $$I_{f}\left(x,y\right)=\frac{1}{W\left(x,y\right)}\sum_{\left(i,j\right)}I_{\mathrm{clahe}}\left(i,j\right)\cdot \exp\left(-\frac{\left(x-i{)}^{2}+(y-j{)}^{2}\right.}{2\sigma_{s}^{2}}\right).\exp\left(-\frac{(I_{\mathrm{clahe}}(x,y)-I_{\mathrm{clahe}}(i,j){)}^{2}}{2\sigma_{r}^{2}}\right)$$

where $\sigma_{s}$ and $\sigma_{r}$ control spatial and intensity similarity, and $W\left(x,y\right)$ is a normalization factor. This pipeline ensures color constancy, corrects illumination, enhances contrast, and preserves structural details, enabling reliable feature extraction and skin cancer classification.

### 3.2. Hair Artifact Removal Using Frangi–Gabor Fusion and Iterative Inpainting

Hair artifacts were removed using an iterative multi-scale filtering framework combining Frangi vesselness filtering, Gabor texture filtering, morphological refinement, and image inpainting. We let the preprocessed grayscale image be denoted as $I_{g}(x,y)$. Hair structures were enhanced using Frangi filtering, which detects tubular structures via eigenvalues of the Hessian matrix. The vesselness response is given as Equation (4)

(4) $$V_{F}\left(x,y\right)=\mathrm{Frangi}\left(I_{g}\left(x,y\right);\sigma_{1},\sigma_{2},\ldots ,\sigma_{n}\right)$$

where multiple scales of σ capture both fine and thick hairs, with the mask shown in Figure 2f. In parallel, Gabor filtering was applied to capture linear-oriented textures. The response for orientation θ is given by Equation (5)

(5) $$G_{\theta }\left(x,y\right)=I_{g}\left(x,y\right)*K_{\theta }\left(x,y\right)$$

where $K_{\theta }$ is a Gabor kernel. Multi-orientation responses were aggregated using a maximum operation, $G(x,y)={max}_{\theta }G_{\theta }(x,y)$, for θ∈{0°,30°,…,150°}, producing the mask in Figure 2g. Hair candidate regions were extracted via adaptive percentile thresholding as $M_{F}=V_{F}(x,y)>T_{F}$ and $M_{G}=G(x,y)>T_{G}$, where $T_{F}$ and $T_{G}$ are dynamically computed. The final hair mask was obtained as $M=M_{F}\cup M_{G}$. To enhance spatial coherence, morphological closing and dilation were applied, as given by Equation (6)

(6) $$M=\mathrm{MorphClose}\left(M\right),\;M=\mathrm{Dilate}\left(M\right)$$

Detected hair regions were removed using Navier–Stokes-based image inpainting (Telea method): $I_{\mathrm{clean}}=\mathrm{Inpaint}(I_{\mathrm{RGB}},M)$. This process was iteratively repeated to refine hair suppression while preserving lesion structure. The fusion of Frangi and Gabor responses enables robust detection of both curved and linear hair artifacts, producing cleaner lesion boundaries for downstream analysis, as shown in Figure 2h. The proposed fusion-based hair artifact removal strategy contributes to the robustness of the overall framework by effectively eliminating both linear and curved hair artifacts while preserving diagnostically important lesion structures for subsequent analysis.

### 3.3. Proposed CutisNet for Lesion Segmentation

Despite many benefits of dermoscopy, accurate lesion segmentation of dermoscopic images is still a challenge because of the ill-defined boundaries of lesions, skin color differences, and artifacts like hairs, shadows, and illumination inconsistencies. Most of the current segmentation methods are unable to simultaneously maintain fine boundary details and high-level semantic context. More down-sampling will result in a loss of spatial precision and less up-sampling will result in a loss of robustness to real-world variations. Recent studies have also shown that performance deteriorates when a domain shift occurs, which further underscores the importance of developing solid, multi-scale architectures [8].

**Figure 3:**
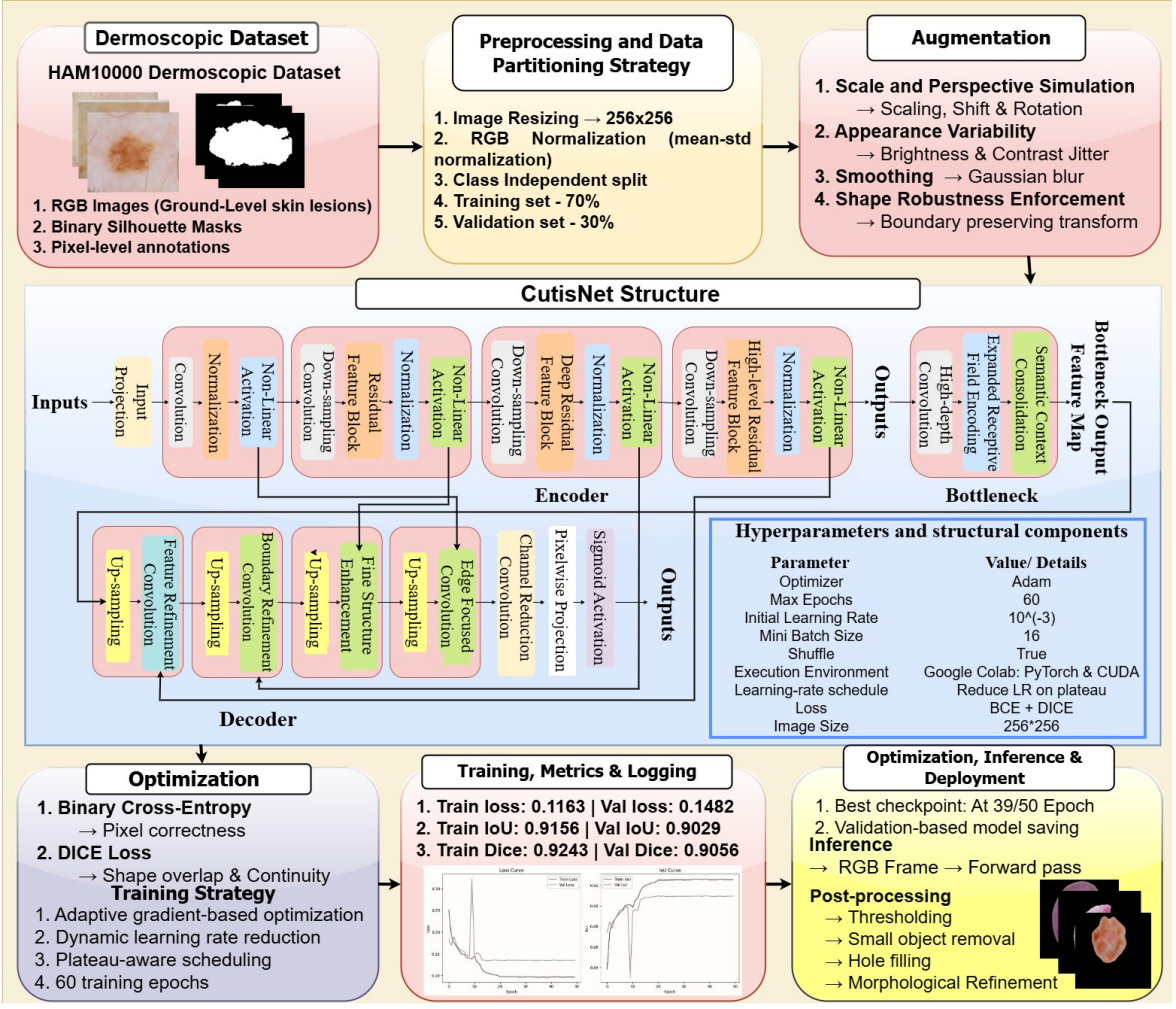
Architecture of CutisNet segmentation model. Overview of the proposed CutisNet architecture for dermoscopic lesion segmentation, illustrating the data preprocessing pipeline, augmentation strategy, hierarchical feature encoding backbone, multi-scale context aggregation, and coarse-to-fine decoder for precise lesion mask generation.

To address these limitations, we propose CutisNet, a specialized encoder–decoder network designed for dermoscopic lesion segmentation. The model is trained using extensive augmentation strategies to simulate real clinical conditions, improving generalization. The architecture is illustrated in Figure 3 and detailed in Table 1. When given an RGB dermoscopic image $I\in R^{H\times W\times 3}$, CutisNet learns a mapping, which is given as Equation (7)

(7) $$M=f_{\theta }(I),M\in \{0,1{\}}^{H\times W}$$

The model is trained on the HAM10000 [14] dataset, which contains dermoscopic RGB images and corresponding expert-annotated binary masks. The dataset is split into 70% training and 30% validation. To enhance robustness, each image–mask pair undergoes augmentation using Equation (8)

(8) $$\left(I^{\prime },M^{\prime })=T_{\mathrm{resize}}\circ T_{\mathrm{flip}}\circ T_{\text{scale-rotate}}\circ T_{\mathrm{blur}}\circ T_{\mathrm{intensity}}(I,M\right)$$

CutisNet follows an encoder–context–decoder structure. The encoder E(⋅) extracts hierarchical features: $F_{e}=E(I)$. A multi-scale context module $B\left(\cdot \right)$ captures global dependencies using parallel dilated convolutions: $F_{c}=B(F_{e})$. Low-level features $F_{l}$ are preserved for spatial refinement: $F_{l}=L(I)$. The decoder $D\left(\cdot \right)$ reconstructs the segmentation by fusing semantic and spatial features using Equation (9)

(9) $$M_{p}=D\left(F_{c},F_{l}\right),M=\sigma \left(M_{p}\right),\sigma (x)=\frac{1}{1+e^{-x}}$$

Training minimizes a hybrid loss by combining Binary Cross-Entropy and Dice loss L, while post-processing refines predictions through thresholding and morphological operations using Equation (10)

(10) $$L\left(p,y\right)=\mathrm{BCE}\left(p,y\right)+\left(1-\frac{2\sum p^{\sigma }y+\epsilon }{\sum p^{\sigma }+\sum y+\epsilon }\right),\;\;\hat{M}=H(S(M_{t})),M_{t}=𝟙(M>0.5)$$

**Figure 4:**
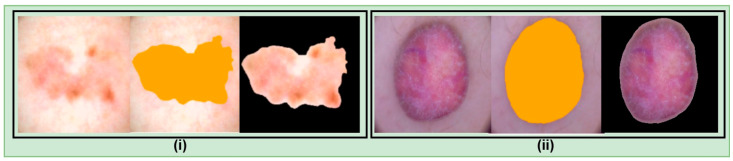
Results of CutisNet segmentation model. Segmentation results of (**i**) PAD-UFES-20 and (**ii**) ISIC (dermatofibroma) datasets: hair-removed images, overlay masks, and segmented images.

The architecture diagram is given in Figure 3, and the segmentation results are presented in Figure 4, demonstrating improved boundary preservation and structural consistency, while the detailed architecture is provided in Table 1.

As shown in Table 2, CutisNet achieves the highest Dice Score and IoU, outperforming the baseline models in segmentation accuracy. Although it has a comparable number of parameters to U-Net++, it delivers superior boundary delineation and robustness, particularly in challenging cases involving hair artifacts and low-contrast lesions. This improvement stems from the effective integration of the EfficientNet-B4 encoder with the DeepLabV3+ decoder, which provides richer multi-scale contextual information.

The proposed CutisNet enhances the robustness of the overall framework by providing accurate and reliable lesion boundary delineation, ensuring high-quality lesion masks for subsequent feature extraction and classification.

### 3.4. Multi-Model Deep Representation Learning

A hybrid deep feature extraction strategy is employed, where a CNN-based MobileNetV2 captures fine-grained local- and texture-level details, while a complementary ViT-based DINO learns global contextual representations. This combination enables robust and discriminative feature learning by effectively modeling both spatial details and long-range dependencies within skin lesions.

#### 3.4.1. CNN-Based Deep Feature Extraction Using MobileNetV2

To learn high-level representations of skin lesions, a pretrained MobileNetV2 [15] network was utilized. Each input image $I\left(x,y,c\right)$ was resized to 224×224 and normalized using Equation (11).

(11) $$I_{\mathrm{norm}}=\frac{I-\mu }{\sigma }$$

where μ and σ are channel-wise parameters. The normalized image was then passed through the network to extract hierarchical features. MobileNetV2 employs depthwise separable convolutions, where standard convolution is decomposed into depthwise and pointwise operations. The depthwise convolution is defined as Equation (12)

(12) $$F_{d}\left(x,y,c\right)=\sum_{i,j}K_{d}\left(i,j,c\right)\cdot I\left(x+i,y+j,c\right)\;\;\;\;\;\;$$

where $K_{d}$ is the depthwise kernel. This is followed by a pointwise 1×1 convolution to combine channel information using Equation (13)

(13) $$F_{p}\left(x,y,k\right)=\sum_{c}K_{p}\left(c,k\right)\cdot F_{d}\left(x,y,c\right)\;\;$$

To capture multi-level representations, activations from the first six expansion blocks were extracted as $A_{l}=f_{l}(I_{\mathrm{norm}}),\;\;l=1,2,\ldots ,6$, where $f_{l}\left(\cdot \right)$ denotes the non-linear transformation at layer l. For each activation tensor $A_{l}\in R^{H_{l}\times W_{l}\times C_{l}}$, channel importance was computed using mean activation, as given by Equation (14)

(14) $$\mu_{k}=\frac{1}{H_{l}\cdot W_{l}}\sum_{x=1}^{H_{l}}\sum_{y=1}^{W_{l}}A_{l}\left(x,y,k\right)$$

**Figure 5:**
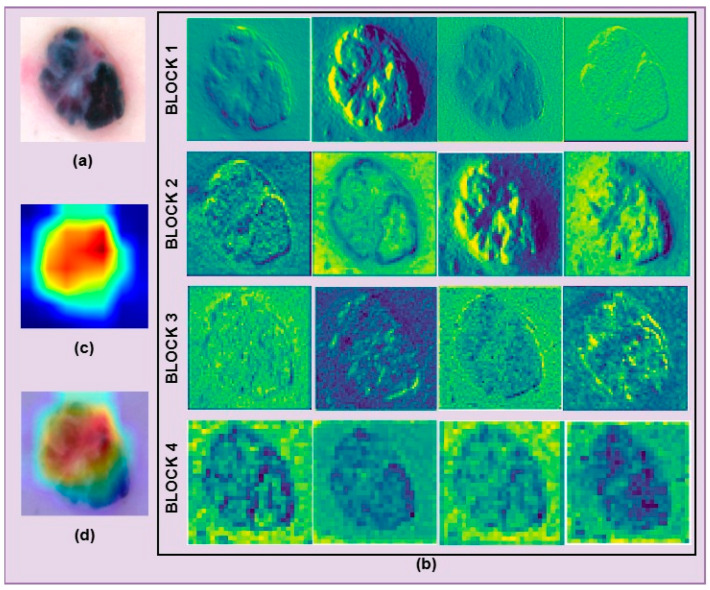
Results of selected blocks and attention maps (using GRAD-CAM) of MobileNetV2 on ISIC dataset (vascular lesion): (**a**) original hair-removed image; (**b**) layer-by-layer results of MobileNet; (**c**) raw attention map; and (**d**) overlay attention map.

The top K channels with the highest $\mu_{k}$ were selected. Each feature map was normalized using Equation (15)

(15) $$A_{l}^{\prime }\left(x,y,k\right)=\frac{A_{l}\left(x,y,k\right)-\mu_{k}}{\sigma_{k}+\epsilon }\;$$

where $\sigma_{k}$ is the standard deviation of the k-th channel, and ϵ ensures numerical stability. These intermediate feature maps, as displayed in Figure 5, capture progressively complex patterns, ranging from low-level edges and textures to high-level lesion-specific structures, enabling MobileNetV2 to efficiently learn discriminative local lesion representations that enhance the robustness of the proposed skin cancer classification framework while maintaining computational efficiency.

#### 3.4.2. Self-Supervised Vision Transformer Feature Extraction Using DINO

To capture global contextual representations, a self-supervised Vision Transformer (ViT-S/8) that was pretrained using DINO was employed. Each hair-removed image $I\left(x,y\right)$ was resized, normalized using ImageNet statistics, and divided into non-overlapping 8×8 patches. The image was converted into token embeddings as $X=[x_{\mathrm{cls}},x_{1},x_{2},\ldots ,x_{N}]$, where $x_{\mathrm{cls}\;}$ is the classification token and N=28×28 is the number of patches, as displayed in Figure 5. Self-attention was computed using Equation (16)

(16) $$A=\mathrm{softmax}\left(\frac{QK^{T}}{\sqrt{d}}\right)\;\;$$

where Q, K, and V are the query, key, and value matrices; and d is the feature dimension. Spatial relevance was obtained using class-to-patch attention $A_{\mathrm{cls}}$, producing a vector of length N, which was reshaped into a spatial grid $A_{\mathrm{grid}}\in R^{28\times 28}$. To improve stability, attention across multiple heads was averaged using Equation (17)

(17) $$A\left(x,y\right)=\frac{1}{H}\sum_{h=1}^{H}A_{h}\left(x,y\right)$$

where H is the number of heads. The resulting attention map was normalized to $\left[0,1\right]$ using Equation (18)

(18) $$A^{\prime }(x,y)=\frac{A(x,y)-min(A)}{max(A)-min(A)}$$

**Figure 6:**
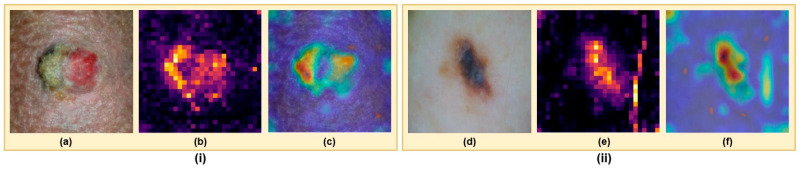
ViT-based DINO results: (**i**) PAD-UFES-20—(**a**) hair-removed image, (**b**) DINO patch, and (**c**) attention map; (**ii**) ISIC (MEL)—(**d**) hair-removed image, (**e**) DINO patch, and (**f**) attention map.

Finally, the map was up-sampled to the original image size using nearest-neighbor interpolation to preserve patch boundaries. The resulting attention maps, as shown in Figure 6, highlight diagnostically relevant lesion regions and capture rich global contextual representations with long-range feature dependencies, enabling the self-supervised ViT-based DINO to complement the local texture information extracted by MobileNetV2 and enhance the robustness of the proposed skin cancer classification framework.

### 3.5. Multi-Strategy Keypoint-Based Feature Extraction

SIFT extracts scale- and rotation-invariant keypoints, while superpixel-based contour analysis captures boundary-aware keypoints, providing complementary structural information.

#### 3.5.1. Superpixel-Based Centroid and Contour Keypoint Extraction

**Figure 7:**
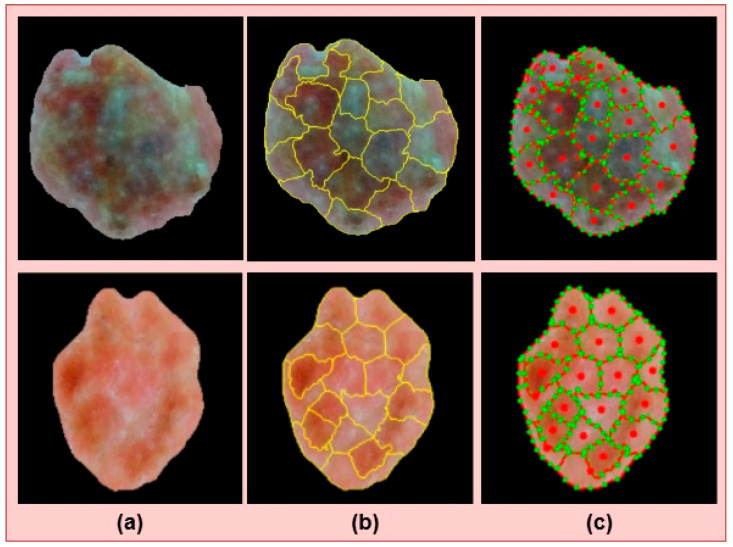
Superpixel-based centroid and contour keypoints: (**a**) segmented lesion images; (**b**) superpixel-based divisions; and (**c**) centroids (red) and contour points (green) of PAD-UFES-20 (top row) and ISIC (MEL) (bottom row).

To capture local structural patterns of skin lesions, the segmented lesion image was partitioned into N superpixels $S_{i}$ using the SLIC algorithm, which clusters pixels based on color and spatial proximity by minimizing the distance metric using Equation (19)

(19) $$D=\sqrt{d_{c}^{2}+{\left(\frac{m}{S}\right)}^{2}d_{s}^{2}}$$

where $d_{c}$ is the color distance, $d_{s}$ is the spatial distance, S is the nominal superpixel size, and m is a compactness parameter controlling shape regularity. For each superpixel $S_{i}$, as shown in Figure 7b, contours $C_{i}$ were extracted using boundary detection. To reduce dimensionality while preserving structural information, keypoints $P_{ij}$ were sampled evenly along each contour by employing Equation (20)

(20) $$P_{ij}=C_{i}\left[\mathrm{round}\left(j\cdot \frac{|C_{i}|}{n}\right)\right],j=0,1,\ldots ,n-1$$

where $|C_{i}|$ is the total number of contour points and n is the number of sampled points per contour. Additionally, the centroid of each superpixel was computed using image moments, as given by Equation (21)

(21) $$\left(x_{c},y_{c}\right)=\left(\frac{M_{10}}{M_{00}},\frac{M_{01}}{M_{00}}\right),M_{pq}=\sum_{x}\sum_{y}x^{p}y^{q}\cdot 1_{S_{i}}\left(x,y\right)$$

where $1_{S_{i}}\left(x,y\right)$ is the indicator function. This process yields superpixels, contour-based keypoints, and centroids, as displayed in Figure 7c. The extracted superpixel centroid and contour keypoints contribute complementary structural and boundary information by preserving lesion geometry and spatial organization, thereby enhancing the robustness of the proposed framework and improving its feature discrimination for skin lesion classification.

#### 3.5.2. SIFT Keypoint-Based Feature Extraction

**Figure 8:**
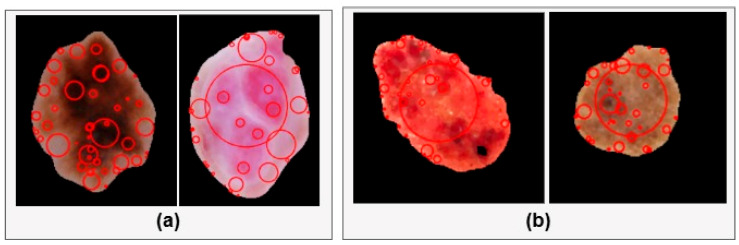
SIFT results on segmented lesions: (**a**) ISIC; (**b**) PAD-UFES-20.

To capture scale- and rotation-invariant local features from skin lesions, the Scale-Invariant Feature Transform (SIFT) algorithm was applied to detect keypoints and compute descriptors. SIFT identifies extrema in the Difference-of-Gaussians (DoGs) scale space defined as Equation (22)

(22) $$D\left(x,y,\sigma \right)=\left(G\left(x,y,k\sigma \right)-G\left(x,y,\sigma \right)\right)*I\left(x,y\right)$$

where $G\left(x,y,\sigma \right)$ is a Gaussian kernel of standard deviation σ, k is a multiplicative scale factor, and ∗ denotes convolution. Keypoints are identified as local extrema of $D\left(x,y,\sigma \right)$ across space and scale. Each keypoint is assigned a dominant orientation θ based on local image gradients using Equation (23)

(23) $$m\left(x,y\right)=\sqrt{\left(I_{x}{)}^{2}+(I_{y}{)}^{2}\right.},\theta \left(x,y\right)=\arctan2\left(I_{y},I_{x}\right)$$

where $I_{x}$ and $I_{y}$ are image gradients in the x and y directions. For each keypoint, a 128-dimensional descriptor is formed from histograms of gradient orientations within a local neighborhood, providing robustness to illumination and rotation. These SIFT features, as displayed in Figure 8, effectively encode edges, corners, and blob-like structures to generate scale- and rotation-invariant local descriptors, improving robustness against variations in lesion size, orientation, and imaging conditions while providing highly discriminative representations for skin lesion classification.

### 3.6. Hybrid Texture Feature Representation

GLRLM, Tamura, and Gabor features are combined to capture run-length statistics, perceptual texture, and frequency-based patterns for comprehensive lesion texture representation.

#### 3.6.1. Texture Analysis Using Gray-Level Run-Length Matrix (GLRLM)

To extract fine-grained texture information from dermoscopic segmented images, each image was first converted to grayscale $I\left(x,y\right)\in \left[0,255\right]$ using the standard luminance formula of I(x,y)=0.2989R(x,y)+0.5870G(x,y)+0.1140B(x,y), where R,G,B are the color channels. The grayscale image was quantized into L levels using Equation (24)

(24) $$I_{q}(x,y)=\left\lfloor \frac{I(x,y)}{256}\cdot L\right\rfloor$$

A w×w sliding window with reflective padding was applied to compute the Gray-Level Run-Length Matrix (GLRLM) [16] of P(i,j), where i is the gray level and j is the run length, counting horizontal runs of length j at gray levels i. From the GLRLM, five texture descriptors were extracted. Short Run Emphasis (SRE) and Long Run Emphasis (LRE) capture short and long texture structures by using Equation (25)

(25) $$SRE=\frac{\sum_{i}\sum_{j}P(i,j)/j^{2}}{\sum_{i}\sum_{j}P(i,j)},LRE=\frac{\sum_{i}\sum_{j}P(i,j)\cdot j^{2}}{\sum_{i}\sum_{j}P(i,j)}$$

Gray-Level Non-Uniformity (GLN) and Run-Length Non-Uniformity (RLN) measure intensity and run variability by using Equation (26)

(26) $$GLN=\frac{\sum_{i}(\sum_{j}P(i,j){)}^{2}}{\sum_{i}\sum_{j}P(i,j)},RLN=\frac{\sum_{j}(\sum_{i}P(i,j){)}^{2}}{\sum_{i}\sum_{j}P(i,j)}$$

Run Percentage (RP) reflects the run density, and is mathematically expressed as Equation (27). Pixel-wise feature maps were generated using sliding windows and normalized via min–max scaling using Equation (27)

(27) $$RP=\frac{\sum_{i}\sum_{j}P(i,j)}{L\cdot R},\;F_{norm}=\frac{F-F_{min}}{F_{max}-F_{min}}$$

**Figure 9:**
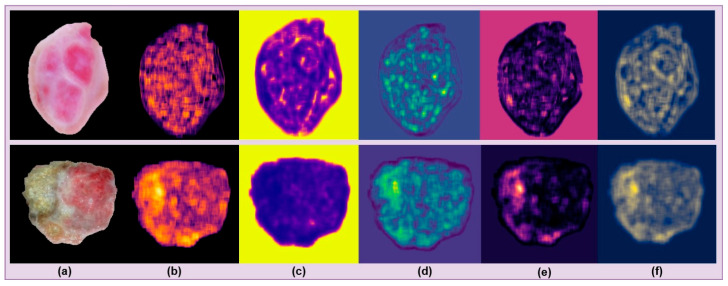
GLRLM outputs: (**a**) segmented lesions; (**b**) SRE; (**c**) LRE; (**d**) GLN; (**e**) RLN; and (**f**) RP of ISIC (VASC) (top row) and PAD-UFES-20 (bottom row).

These normalized GLRLM maps, as displayed in Figure 9, capture local texture variations in skin lesions and serve as discriminative features for classification. GLRLM features contribute complementary statistical texture information by capturing gray-level run-length characteristics associated with lesion homogeneity and structural heterogeneity.

#### 3.6.2. Gabor Filter-Based Texture Analysis

**Figure 10:**
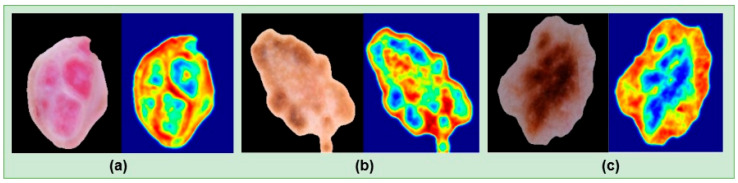
Gabor-based texture response on ISIC dataset: (**a**) VASC, (**b**) MEL, and (**c**) NV.

Gabor filters, which combine Gaussian envelopes with sinusoidal plane waves, were applied to the enhanced grayscale image. A 2D Gabor filter $G\left(x,y;\lambda ,\theta ,\sigma ,\gamma \right)$, at wavelength λ, orientation θ, Gaussian standard deviation σ, and spatial aspect ratio γ, is defined as Equation (28)

(28) $$G(x,y)=exp(-\frac{x^{\prime 2}+\gamma^{2}y^{\prime 2}}{2\sigma^{2}})\cdot cos(2\pi \frac{x^{\prime }}{\lambda })$$

where  $x^{\prime }=x\cos\theta +y\sin\theta \;and{\;y}^{\prime }=-x\sin\theta +y\cos\theta .$ The filter was applied at multiple orientations θ∈{0,π/4,π/2,3π/4} and wavelengths corresponding to spatial frequencies $f\in \left\{0.05,0.1,0.2\right\}$ using 2D convolution as $R_{\lambda ,\theta }(x,y)=(I*G_{\lambda ,\theta })(x,y),$ where ∗ denotes convolution. The Gabor energy map was then computed using Equation (29), as depicted in Figure 10, by summing the absolute values of all filter responses.

(29) $$E\left(x,y\right)=\sum_{\lambda }\sum_{\theta }|R_{\lambda ,\theta }\left(x,y\right)|$$

This was then normalized to the range of $\left[0,255\right]$ for visualization by using Equation (30)

(30) $$E_{\mathrm{norm}}\left(x,y\right)=\frac{E\left(x,y\right)-E_{min}}{E_{max}-E_{min}}\cdot 255$$

where $E_{min}$ and $E_{max}$ are the minimum and maximum values of E(x,y), respectively. These Gabor energy maps encode directional and frequency-specific texture patterns of skin lesions, capturing structural variations, such as streaks, ridges, and spots. Gabor features contribute multi-scale and multi-orientation texture representations that effectively characterize directional lesion patterns and fine surface textures, enhancing feature discrimination.

#### 3.6.3. Tamura Texture Feature Extraction

**Figure 11:**
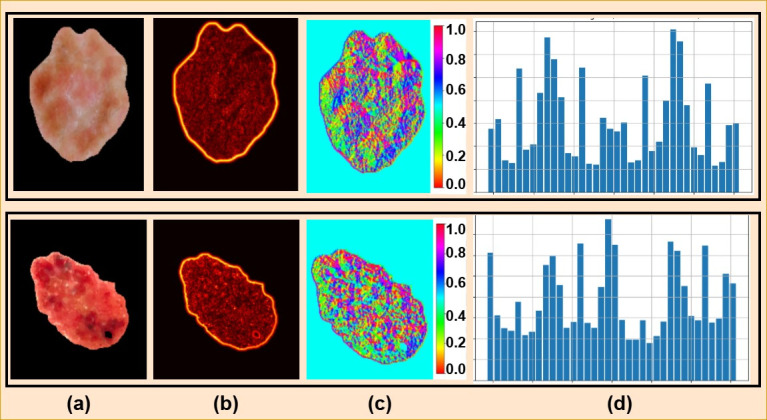
Tamura texture outputs: (**a**) lesion images; (**b**) Tamura contrast map; (**c**) directionality map; and (**d**) directionality histogram on ISIC (top row) and PAD-UFES-20 (bottom row) datasets.

To characterize skin lesion textures, the input image was first converted to grayscale, and Tamura texture descriptors were computed for contrast and directionality. The contrast map, as shown in Figure 11b, captures intensity variation using standard deviation σ, mean μ, and normalized fourth central moment $\alpha_{4}$, as defined by Equation (31)

(31) $$\alpha_{4}=\frac{1}{HW}\sum \frac{\left(I-\mu {)}^{4}\right.}{\sigma^{4}},\;\mathrm{Contrast}=\frac{\sigma }{\alpha_{4}^{1/4}}$$

Figure 11c shows the directionality map that captures edge orientation consistency. Image gradients are computed using Sobel operators, as given by Equation (32)

(32) $$G_{x}=\frac{\partial I}{\partial x},G_{y}=\frac{\partial I}{\partial y},M=\sqrt{G_{x}^{2}+G_{y}^{2}},\theta ={tan}^{-1}\left(\frac{G_{y}}{G_{x}}\right)$$

A magnitude-weighted histogram of θ is constructed, and directionality is defined as the variance of this histogram, i.e., $\mathrm{Var}\left(\mathrm{Histogram}\left(\theta ,M\right)\right),$ as displayed in Figure 11d. These Tamura-based contrast and directionality descriptors provide a compact representation of local intensity variation and edge orientation patterns in skin lesions, supporting effective classification.

### 3.7. Morphological and Geometric Feature Extraction

To effectively characterize the structural properties of skin lesions, a set of geometric and shape-based descriptors is extracted. These features capture global morphology, invariant shape patterns, and symmetry characteristics, providing complementary information for robust lesion analysis.

#### 3.7.1. Ellipse-Based Geometric Feature Extraction

**Figure 12:**
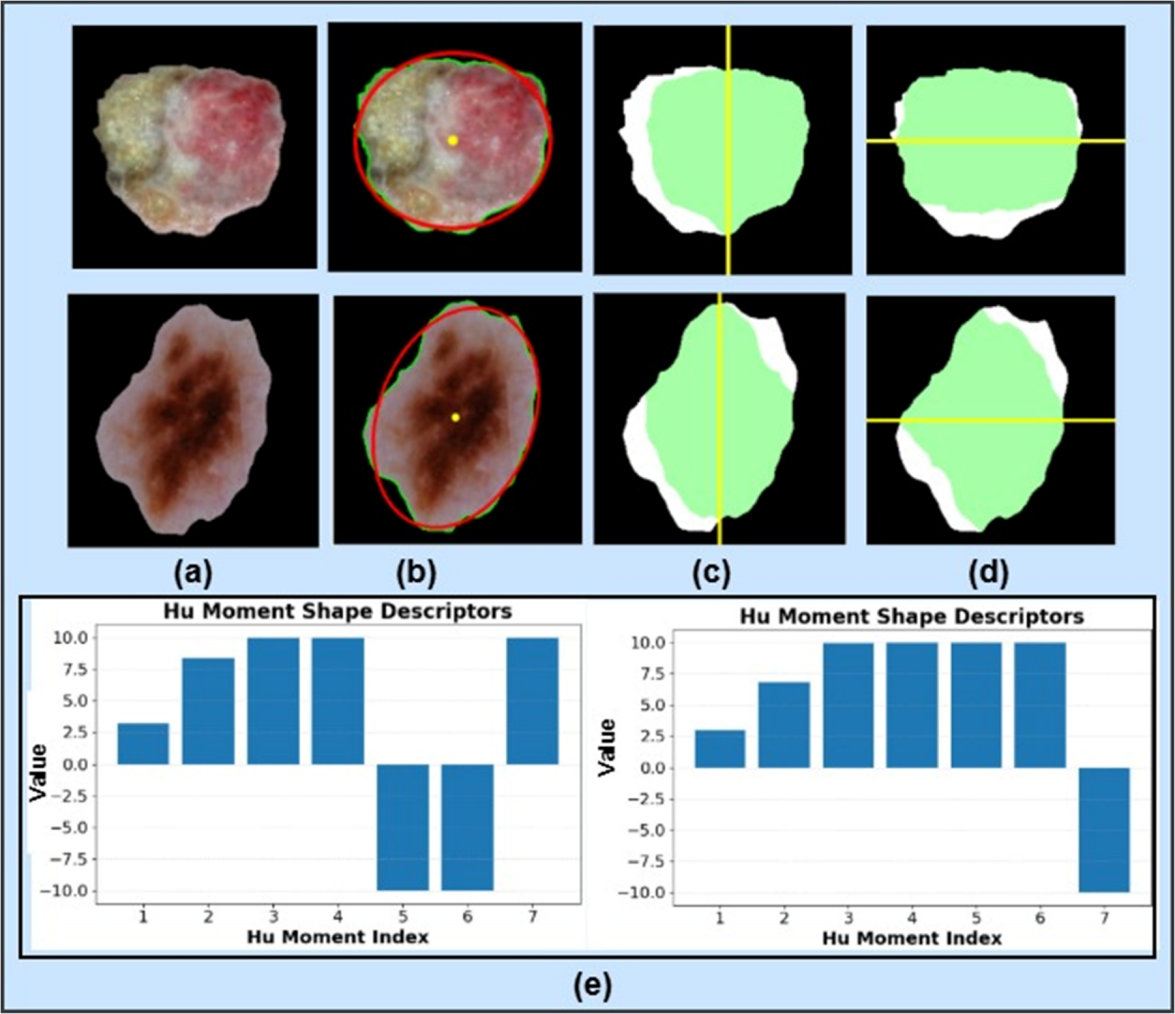
Geometric and shape-based features: (**a**) segmented lesion images; (**b**) ellipse-fitting results; (**c**) vertical symmetry; (**d**) horizontal symmetry; and (**e**) Hu moments on PAD-UFES-20 (**left**) and ISIC (NV) (**right**) datasets.

To characterize global lesion shape, the dermoscopic image $I\left(x,y\right)$ was converted to grayscale $I_{g}\left(x,y\right)$ and smoothed using Gaussian filtering with $I_{s}(x,y)=G_{\sigma }(x,y)*I_{g}(x,y)$, where $G_{\sigma }$ is a Gaussian kernel and ∗ denotes convolution. A binary lesion mask B(x,y) was obtained via Equation (33)

(33) $$B(x,y)=\left\{\begin{array}{cc} 1, & I_{s}(x,y)>0\\ 0, & \mathrm{otherwise} \end{array}\right.\;$$

ensuring that the lesion region is represented by white pixels. The largest contour C was extracted and fitted with an ellipse using least squares, yielding the center $\left(x_{c}{,y}_{c}\right)$, major axis a, minor axis b, and orientation θ. Ellipticity was defined as ϵ=a/b. The resulting mask is given in Figure 12b. These geometric features (a,b,θ,ϵ) provide a compact representation of lesion shape, capturing elongation and orientation characteristics for distinguishing benign and malignant lesions. Ellipse fitting contributes geometric characterization of lesion morphology by providing robust shape descriptors that complement texture and deep feature representations.

#### 3.7.2. Symmetry-Based Shape Feature Extraction

To quantify structural asymmetry, binary lesion masks $B\left(x,y\right)\in \left\{0,1\right\}$ were used, where non-zero pixels represent the lesion. Symmetry was evaluated along vertical and horizontal axes by comparing the mask with its flipped versions. For vertical symmetry, the reflected mask $B_{v}\left(x,y\right)$ is defined as Equation (34) and displayed in Figure 12c.

(34) $$B_{v}\left(x,y\right)=B\left(x,W-1-y\right)$$

where W is the image width. The overlap $O_{v}$ and vertical symmetry score $S_{v}$ are computed using Equation (35)

(35) $$O_{v}(x,y)=B(x,y)\cdot B_{v}(x,y),\;S_{v}=\frac{\sum_{x,y}O_{v}(x,y)}{\sum_{x,y}B(x,y)}$$

For horizontal symmetry, the flipped mask $B_{h}\left(x,y\right)$ is given as Equation (36), as shown in Figure 12d.

(36) $$B_{h}\left(x,y\right)=B\left(H-1-x,y\right)$$

where H is the image height. The overlap $O_{h}$ and symmetry score $S_{h}$ were computed using Equation (37)

(37) $$O_{h}\left(x,y\right)=B\left(x,y\right)\cdot B_{h}\left(x,y\right),\;S_{h}=\frac{\sum_{x,y}O_{h}(x,y)}{\sum_{x,y}B(x,y)}$$

The scores $S_{v}$ and $S_{h}\in [0,1]$ indicate greater symmetry with higher values. Symmetry analysis contributes clinically relevant morphological information by quantifying lesion asymmetry, an important indicator for distinguishing benign and malignant skin lesions.

#### 3.7.3. Hu Moment Shape Feature Extraction

To capture the global shape characteristics of skin lesions, binary lesion masks $B\left(x,y\right)$ are used.

The raw spatial moments of the binary mask were computed using Equation (38)

(38) $$m_{pq}=\sum_{x}\;\sum_{y}x^{p}y^{q}\;B(x,y),p,q=0,1,2,\ldots$$

From these, the central moments are defined as Equation (39)

(39) $$\mu_{pq}=\sum_{x}\sum_{y}(x-\bar{x}{)}^{p}(y-\bar{y}{)}^{q}\;B(x,y),\bar{x}=\frac{m_{10}}{m_{00}},\bar{y}=\frac{m_{01}}{m_{00}}$$

The normalized central moments are given by Equation (40)

(40) $$\eta_{pq}=\frac{\mu_{pq}}{\mu_{00}^{\gamma }},\;\gamma =\frac{p+q}{2}+1$$

Using these, the seven Hu invariant moments $H_{1},\ldots ,H_{7}$ were computed, which are invariant to translation, scale, and rotation. For numerical stability, a logarithmic transformation was applied using Equation (41)

(41) $$H_{i}^{\log}=-\mathrm{sign}(H_{i})\cdot {log}_{10}(|H_{i}|+\epsilon ),i=1,\ldots ,7$$

where $\epsilon =10^{-10}$ avoids singularities. The centroid of the lesion was also computed as $\left(\bar{x},\bar{y}\right)$. These Hu moments, graphically represented in Figure 12e, provide a compact and rotation-invariant descriptor of lesion shape, complementing other geometric and texture features for robust skin cancer classification.

### 3.8. Feature Fusion Strategy

The proposed framework integrates heterogeneous representations by combining deep embeddings and handcrafted descriptors into a unified feature vector. We let $F_{d}^{\left(1\right)}\in R^{p}$ and $F_{d}^{\left(2\right)}\in R^{q}$ denote deep features from the ViT-based DINO and MobileNetV2, respectively. Texture features $F_{t}$ are extracted using Tamura descriptors, GLRLM, and Gabor responses, while shape features $F_{s}$ include Hu moments, ellipse parameters, and symmetry descriptors. Keypoint features $F_{k}$ are derived from SIFT, superpixel centroids, and contour-based distributions. All feature vectors are first normalized using min–max scaling and computed by Equation (42)

(42) $$\hat{F}=\frac{F-min(F)}{max(F)-min(F)}$$

The normalized features are then fused at the feature level using Equation (43), where ∥ denotes concatenation.

(43) $$F_{f}=\left[{\hat{F}}_{d}^{\left(1\right)}\;\;∥\;\;{\hat{F}}_{d}^{\left(2\right)}\;\;∥\;\;{\hat{F}}_{t}\;\;∥\;\;{\hat{F}}_{s}\;\;∥\;\;{\hat{F}}_{k}\right]$$

The deep and handcrafted features are normalized using min–max scaling and concatenated to form a fused feature vector of 5313 dimensions. Gray Wolf Optimization (GWO) is then applied to select the most discriminative features, resulting in a final optimized feature vector of 1856 dimensions, as given in Table 3.

### 3.9. Gray Wolf Optimization for Feature Selection and Refinement

To enhance discriminative power and reduce redundancy in the fused feature space $F_{f}$, Gray Wolf Optimization (GWO) is employed for adaptive feature weighting and selection. Each candidate solution represents a weight vector as $w=[w_{1},w_{2},\ldots ,w_{n}]$, where $w_{i}\in [0,1]$ denotes feature importance. The objective is to minimize classification loss $J\left(w\right)$ with regularization computed using Equation (44). GWO is guided by three leaders α,β and δ (best solutions), while the remaining wolves update their positions using Equation (44)

(44) $$J(w)=L(f(F_{f}⊙w))+\lambda ∥w{∥}_{2}^{2},\;D=|C\cdot X_{p}-X|,X(t+1)=X_{p}-A\cdot D$$

where $X_{p}$ is the best solution. The coefficients are given by Equation (45)

(45) $$A=2a\cdot r_{1}-a,C=2\cdot r_{2}$$

where a decreases linearly from 2 to 0, and $r_{1},r_{2}\in [0,1]$ ensuresthe exploration–exploitation balance. The optimal weights $w^{*}$ are obtained from α,β,δ, producing refined features, as calculated by Equation (46)

(46) $$F_{opt}=F_{f}⊙w^{*}$$

This optimization enhances class separability by retaining informative features while suppressing redundant and noisy components, improving the performance of the downstream GNN–CNN classifier.

Gray Wolf Optimization (GWO) was selected for feature selection because the proposed framework generates a high-dimensional fused feature space by integrating deep and handcrafted features, which may contain redundant and correlated information. GWO effectively identifies the most discriminative feature subset while preserving complementary information. Compared with Genetic Algorithm (GA), which relies on computationally intensive crossover and mutation operations, and Particle Swarm Optimization (PSO), which may converge prematurely, GWO achieves a better exploration–exploitation balance through its hierarchical leadership mechanism. Unlike Ant Colony Optimization (ACO), Differential Evolution (DE), and Salp Swarm Algorithm (SSA), GWO requires fewer control parameters and provides stable convergence with lower computational complexity. By reducing redundant features while retaining lesion-specific texture, shape, and structural information, GWO improves classifier generalization and enhances the accuracy and efficiency of the proposed skin cancer classification framework. Table 4 shows the comparison of effect of various optimizers on the overall accuracy of the model.

### 3.10. Hybrid GNN–CNN Classification Framework

The optimized feature vector $F_{opt}\in R^{n}$ is classified using a hybrid GNN–CNN architecture to capture both relational and local feature patterns. First, $F_{opt}$ is represented as a graph G=(V,E), where each node $v_{i}$ corresponds to feature $f_{i}$, and edges $e_{ij}$ are defined using similarity, as given by Equation (47). In the GNN, node embeddings are updated via message passing using Equation (47)

(47) $$e_{ij}=\exp\left(-\frac{∥f_{i}-f_{j}{∥}^{2}}{\sigma^{2}}\right),\;h_{i}^{\left(l+1\right)}=\sigma \left(\sum_{j\in N(i)}\frac{1}{c_{ij}}W^{\left(l\right)}h_{j}^{\left(l\right)}\right)$$

In parallel, $F_{opt}$ is reshaped and processed by a 1D CNN to capture local correlations using Equation (48). The GNN and CNN outputs are fused by concatenation using Equation (48)

(48) $$z^{\left(k\right)}=\phi \left(W^{\left(k\right)}*F_{opt}+b^{\left(k\right)}\right),{\;\;\;\;\;\;\;F}_{hybrid}=\left[F_{GNN}∥F_{CNN}\right]$$

Finally, classification is performed using a fully connected layer with softmax, as given by Equation (49)

(49) $$\hat{y}=\mathrm{softmax}(W_{s}F_{hybrid}+b_{s})$$

The hybrid GNN–CNN classifier contributes complementary local feature learning and graph-based relational modeling, enabling more robust discrimination of visually similar skin lesion categories and improving multi-class classification performance. The complete implementation details, including the network configuration, feature dimensions, training hyperparameters, Gray Wolf Optimization parameters, and hardware/software environment, are summarized in Table 5.

## 4. Results

### 4.1. Dataset Description

The proposed framework was evaluated on five publicly available skin lesion datasets containing RGB dermoscopic and clinical images with diverse lesion categories and acquisition conditions. The PAD-UFES-20 [17] dataset contains 2298 RGB clinical images from six classes: melanoma, basal cell carcinoma, squamous cell carcinoma, actinic keratosis, seborrheic keratosis, and nevus. The ISIC [6] dataset is a large-scale benchmark dataset comprising RGB dermoscopic images with multiple lesion categories acquired under varying imaging conditions. The Derm7pt [18] dataset contains approximately 2000 RGB dermoscopic images. In this work, five classes were utilized: melanoma, nevus, basal cell carcinoma, seborrheic keratosis, and dermatofibroma. The BCN20000 [12] dataset consists of over 19,000 RGB dermoscopic images from eight classes: melanoma, nevus, basal cell carcinoma, actinic keratosis, benign keratosis, dermatofibroma, vascular lesion, and squamous cell carcinoma. The PH^2^ [19] dataset contains 200 high-resolution RGB dermoscopic images categorized into common nevus, atypical nevus, and melanoma classes.

### 4.2. Training, Validation, and Testing Procedure

The training, validation, and testing procedure adopted in this study is described below, including the data splitting strategy, patient-level data handling, and hyperparameter tuning protocol.

• Data Splitting: We performed five-fold stratified cross-validation with an 80/20 train–validation split.
• Patient-Level Separation: We did not apply patient-level splitting in the current study. This was primarily because most of the publicly available datasets used (such as ISIC, Derm7pt, BCN20000, and PH2) do not provide consistent and reliable patient-level metadata or clear linkage between images and patients. Therefore, data splitting was performed at the image level across all datasets.
• Hyperparameter Tuning: Hyperparameters were tuned solely on the validation set of the first fold, and the selected configuration was fixed for all subsequent folds to minimize data leakage.

### 4.3. Computational Cost Analysis

Table 6 summarizes the computational cost of each module in the proposed framework. While the ViT-based DINO incurs the highest computational complexity and the hair removal stage requires the longest execution time, MobileNetV2 and the handcrafted feature extraction methods maintain relatively low computational overhead. Overall, the proposed framework achieves an effective balance between computational efficiency and robust skin cancer classification performance.

### 4.4. Confusion Matrices

**Figure 13:**
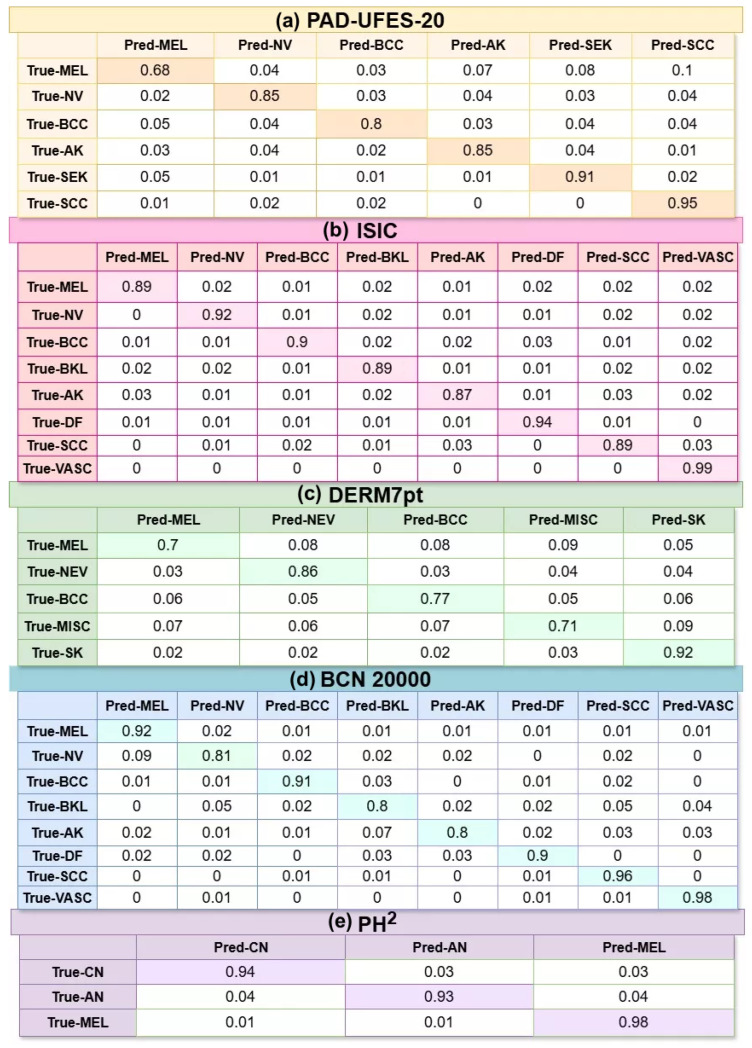
Confusion matrices: (**a**) PAD-UFES-20; (**b**) ISIC; (**c**) Derm7pt; (**d**) BCN20000; and (**e**) PH^2^.

Figure 13 shows the confusion matrices on the five datasets. The PAD-UFES-20 confusion matrix shows strong recognition for SCC (95%), SEK (91%), and AK (85%). MEL achieved lower accuracy (68%) due to visual similarity and overlapping texture patterns with SEK and SCC, while NV and BCC attained 85% and 80% accuracy, respectively. For the ISIC dataset, high multi-class performance was achieved across all classes, with VASC (99%) and DF (94%) showing the highest accuracy. Minor misclassification occurred among MEL, BKL, AK, and SCC due to similar pigmentation, irregular borders, and lesion morphology. The Derm7pt dataset achieved strong recognition for SK (92%) and NEV (86%), whereas MEL (70%) and MISC (71%) showed higher confusion because of heterogeneous dermoscopic structures and overlapping visual characteristics. BCC achieved 77% accuracy with limited cross-class confusion. The BCN20000 confusion matrix demonstrated strong classification for VASC (98%), SCC (96%), MEL (92%), and BCC (91%). Misclassification among NV, BKL, and AK was mainly caused by high intra-class variability and similar lesion appearance. The PH^2^ dataset showed highly reliable classification, with MEL, CN, and AN achieving 98%, 94%, and 93% accuracy, respectively. Minor confusion between CN and AN resulted from subtle visual differences in lesion boundaries and pigmentation.

### 4.5. Results and Discussion

The proposed framework, as shown in Table 7, achieved 88.0% accuracy on the PAD-UFES-20 dataset, with precision, recall, and F1-score of 0.845, 0.84, and 0.841, respectively. On the ISIC dataset, the model achieved 94.5% accuracy, with precision, recall, and F1-score of 0.907, 0.91, and 0.911, demonstrating strong multi-class performance. The Derm7pt dataset achieved 83.6% accuracy with precision, recall, and F1-score of 0.792, 0.79, and 0.792, respectively. For the BCN20000 dataset, the framework obtained 93.2% accuracy, with precision, recall, and F1-score of 0.877, 0.885, and 0.88. The PH^2^ dataset achieved the highest performance, with 96.7% accuracy, 0.943 precision, 0.95 recall, and 0.947 F1-score. All reported results represent the mean ± standard deviation obtained from five-fold stratified cross-validation (80/20 train–test split). The 95% confidence intervals were calculated as mean ± 1.96 × (SD/√5). The relatively low standard deviations across all datasets demonstrate the robustness and stability of the proposed framework.

Table 7 further demonstrates that model achieved high sensitivity (79.2–94.7%) and specificity (94.8–98.7%), indicating its effectiveness in correctly identifying skin lesion classes while minimizing false-positive predictions. Furthermore, the balanced accuracy values (87.0–96.2%) confirm reliable performance under class-imbalanced conditions. Although PAD-UFES-20 exhibited comparatively lower sensitivity due to the challenging nature of melanoma classification and dataset imbalance, the framework maintained competitive performance on this dataset and consistently strong results across all other benchmarks. The low standard deviations and narrow 95% confidence intervals further demonstrate the robustness and stability of the proposed framework.

**Figure 14:**
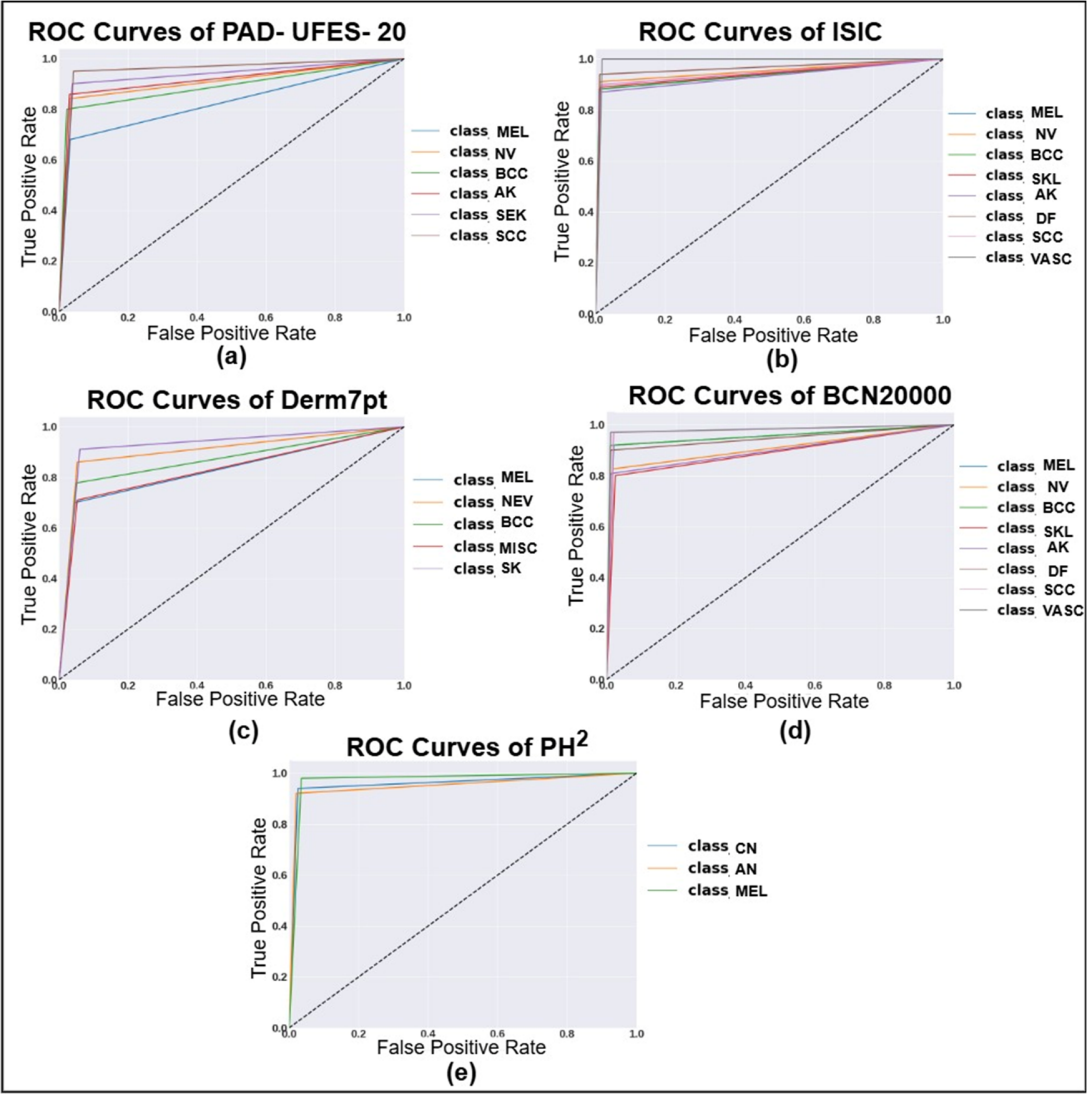
ROC curves of (**a**) PAD-UFES-20; (**b**) ISIC; (**c**) Derm7pt; (**d**) BCN20000; and (**e**) PH^2^.

**Figure 15:**
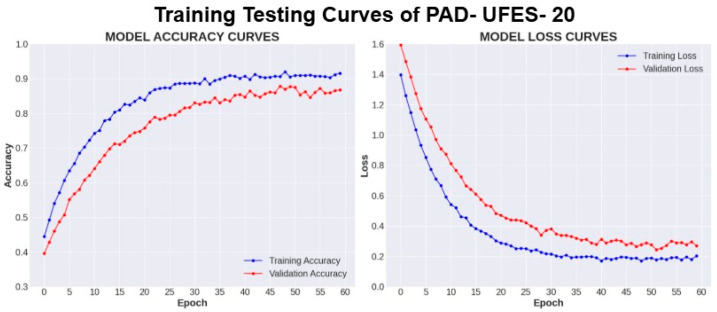
Training and testing curves of model on PAD-UFES-20.

The ROC curve analysis for all datasets is presented in Figure 14. The PAD-UFES-20 dataset demonstrates effective class separability, with slight performance degradation for MEL due to overlap with visually similar lesion categories. For the ISIC dataset, the ROC curves remain close to the top-left region, indicating high sensitivity and specificity across all classes. The Derm7pt dataset exhibits comparatively lower ROC performance for the MEL and MISC classes because of heterogeneous dermoscopic structures and inter-class similarity. The ROC curves of the BCN20000 dataset indicate strong discriminative capability, with minor variations among the NV, BKL, and AK classes caused by similar lesion morphology. The PH^2^ dataset achieved near-ideal ROC characteristics, demonstrating highly reliable lesion discrimination and melanoma detection performance. The training and testing curves of PAD-UFES-20 are shown in Figure 15.

Although the proposed framework achieved an overall competitive performance on the PAD-UFES-20 dataset (88.0%), melanoma exhibited comparatively lower recognition than other lesion categories. This can be attributed to the inherent challenges associated with melanoma classification. Melanoma demonstrates high intra-class variability, with substantial differences in color, pigmentation, border irregularity, size, and texture, while early-stage melanomas often closely resemble benign lesions. Moreover, melanoma shares significant visual characteristics with seborrheic keratosis, basal cell carcinoma, and squamous cell carcinoma, resulting in increased inter-class confusion. Another contributing factor is the relatively limited number of melanoma samples in the PAD-UFES-20 dataset, which restricts the model’s ability to learn sufficiently representative melanoma-specific patterns. Nevertheless, despite these challenges, the proposed framework achieved higher overall accuracy than several existing state-of-the-art methods on the same dataset and demonstrated consistently strong performance across all five benchmark datasets. Future work will focus on improving melanoma sensitivity by leveraging diffusion-based synthetic data generation and adaptive class-balanced focal loss to mitigate dataset imbalance, thereby improving the framework’s robustness and clinical applicability.

### 4.6. Ablation Study

In Table 8, the ablation study validates the effectiveness of each module in the proposed framework. The full model, integrating preprocessing, deep features, lesion keypoints, textural descriptors, and shape-based features, achieves the highest accuracies of 88.0%, 94.5%, 83.6%, 93.2%, and 96.7% on PAD-UFES-20, ISIC, Derm7pt, BCN20000, and PH^2^, respectively. Removing preprocessing reduces robustness, while excluding MobileNetV2 and ViT-based DINO features causes the largest performance decline, highlighting the importance of deep semantic representations. Likewise, removing lesion keypoints, texture descriptors, and shape-based features consistently decreases accuracy, confirming the complementary role of structural, textural, and morphological information in skin lesion classification.

### 4.7. Comparative Analysis

The proposed model consistently outperforms state-of-the-art methods across multiple datasets, as shown in Table 9. On PAD-UFES-20, prior works have reported accuracies, such as Khurshid et al. (0.851), Tang et al. (0.830), and Uliana and Krohling (0.6457), while the proposed model achieves 88.0%. For ISIC, existing methods have included Moodi et al. (0.7142), Pacal et al. (0.9254), Ozdemir et al. (93.60%), and Yang et al. (92.791%). The proposed model attains 94.5%, outperforming them all. On Derm7pt, methods such as Xu et al. (0.813), Tang et al. (0.785), and Bi et al. (0.699) have shown lower performance, whereas the proposed model reaches 83.6%. For BCN20000, prior results have included Agarwal et al. (91.17%), Joshi et al. (92%), and Gunaratne et al. (91.9%), while the proposed model improves to 93.2%. On PH^2^, existing methods such as Nawaz et al. (95.6%) and Toprak et al. (94.44%) are surpassed by the proposed model, which achieves 96.7%.

### 4.8. Workflow Integration

The proposed framework is intended to function as a computer-aided decision support system rather than a replacement for dermatologist expertise. In a typical clinical workflow, dermoscopic or clinical skin lesion images would first undergo automated preprocessing, artifact removal, and lesion segmentation, followed by feature extraction and classification. The predicted lesion category, together with attention visualizations and interpretable handcrafted feature descriptors, can provide clinicians with complementary information to support diagnostic decision-making. Final diagnosis and treatment planning would remain under the supervision of dermatologists, who can integrate the model’s predictions with patient history, physical examination, and other clinical findings. Future work will focus on prospective clinical validation, integration with hospital information systems, and evaluation in real-world clinical settings before considering routine clinical deployment.

## 5. Limitations

While the proposed framework demonstrates strong performance across multiple benchmark datasets, several limitations should be acknowledged. First, patient-level splitting was not implemented in the current study due to the unavailability of consistent and reliable patient metadata in most public datasets (e.g., ISIC, Derm7pt, BCN20000, and PH2), where images often lack clear patient linkage. Consequently, data splitting was performed at the image level, which may introduce a minor risk of data leakage in cases where multiple images from the same patient exist. Furthermore, differences in imaging devices, illumination conditions, patient characteristics, and rare lesion categories may influence the generalizability of the proposed framework in real-world clinical settings. Although the hybrid deep learning architecture demonstrated promising performance, it still lacks robust interpretability mechanisms, making clinical decision explanation and physician trust more challenging. In addition, although Gray Wolf Optimization (GWO) effectively reduced the feature dimensionality from 5313 to 1856 while improving classification performance, it is inherently susceptible to limitations, such as potential premature convergence, which may affect the identification of the globally optimal feature subset.

## 6. Conclusions and Future Work

This study presented a comprehensive hybrid framework for automated skin cancer classification by integrating advanced preprocessing, lesion segmentation, deep learning, handcrafted feature extraction, optimization, and hybrid classification techniques into a unified pipeline. The proposed CutisNet segmentation architecture effectively preserved lesion boundaries and improved robustness under challenging imaging conditions, while the combination of MobileNetV2, self-supervised ViT-based DINO, and uniquely selected handcrafted descriptors enabled comprehensive representation of local textures, structural properties, and global contextual information. Furthermore, the incorporation of Gray Wolf Optimization and a hybrid GNN–CNN classifier significantly enhanced discriminative feature learning and multi-class classification performance. Experimental evaluations demonstrated strong performance across multiple benchmark datasets, achieving accuracies of 88.0% on PAD-UFES-20, 94.5% on International Skin Imaging Collaboration—ISIC, 83.6% on Derm7pt, 93.2% on BCN20000, and 96.7% on PH^2^, thereby confirming the robustness and generalization capability of the proposed framework. Comparative evaluations and ablation studies further validated the contribution of each module toward improved diagnostic accuracy. Future work will focus on improving model interpretability, reducing computational complexity, and incorporating attention-based multimodal learning strategies to enhance real-world clinical applicability and diagnostic reliability. In addition, future studies will prioritize the use of datasets with reliable patient-level annotations to enable patient-level data splitting and further validate the generalizability of the proposed framework in real-world clinical settings. Furthermore, hybrid metaheuristic optimization strategies, such as Gray Wolf Optimization (GWO) combined with Particle Swarm Optimization (PSO) or Genetic Algorithms (GAs), will be investigated to achieve a better exploration–exploitation balance, mitigate premature convergence, and further enhance feature selection performance.

## Figures and Tables

**Table 1:** Architecture of proposed CutisNet.

Stage	Block ID	Module Name	Layer Composition	Input Size	Output Size	Purpose
Input	I	Input Projection	RGB Image	3 × 256 × 256	3 × 256 × 256	Raw dermoscopic input
Encoder	E1	Shallow Feature Extraction	Conv 3 × 3 → BN → SiLU	3 × 256 × 256	48 × 128 × 128	Initial texture and color feature capture
Encoder	E1	Down-Sampling	Strided Conv (stride = 2)	48 × 128 × 128	48 × 128 × 128	Spatial reduction
Encoder	E2	Mobile Inverted Bottleneck	Expansion Conv → Depthwise Conv → SE → Projection Conv	48 × 128 × 128	32 × 128 × 128	Efficient local feature encoding
Encoder	E3	Hierarchical Encoding	MBConv Blocks ×3 (stride 2 in first block)	32 × 128 × 128	56 × 64 × 64	Multi-scale spatial abstraction
Encoder	E4	Mid-Level Representation	MBConv Blocks ×4 (stride 2 in first block)	56 × 64 × 64	160 × 32 × 32	Structural pattern learning
Encoder	E5	High-Level Semantic Encoding	MBConv Blocks ×6 (stride 2 in first block)	160 × 32 × 32	448 × 16 × 16	Deep semantic representation
Context	B	Multi-Scale Context Encoder	Parallel 1 × 1 Conv + 3 × 3 Dilated Conv (rates: 6, 12, 18) → Concatenation → 1 × 1 Conv	448 × 16 × 16	256 × 16 × 16	Multi-scale receptive field expansion
Low-Level	L	Feature Refinement Path	1 × 1 Conv → BN → ReLU	48 × 128 × 128	48 × 128 × 128	Preserves fine spatial details
Decoder	D1	Up-Sampling	Bilinear Up-Sampling (×4)	256 × 16 × 16	256 × 64 × 64	Restores coarse spatial resolution
Decoder	D1	Feature Fusion	Concatenation (with L) → Conv 3 × 3 → BN → ReLU	(256 + 48) × 64 × 64	256 × 64 × 64	Combines semantic + spatial cues
Decoder	D2	Refinement	Conv 3 × 3 → BN → ReLU	256 × 64 × 64	128 × 64 × 64	Feature smoothing and boundary refinement
Output	O	Channel Reduction	1 × 1 Conv	128 × 64 × 64	1 × 64 × 64	Pixel-wise logits
Output	O	Up-Sampling	Bilinear Interpolation (×4)	1 × 64 × 64	1 × 256 × 256	Restores original resolution
Output	O	Activation	Sigmoid	1 × 256 × 256	1 × 256 × 256	Binary lesion probability map

**Table 2:** Quantitative comparison of the proposed CutisNet with established skin lesion segmentation architectures (U-Net, U-Net++, and DeepLabV3+) in terms of Dice Score, IoU (Jaccard Index), model complexity (parameters), and inference time.

Model	Dice Score	IoU (Jaccard)	Parameters (M)	Inference Time (ms)
U-Net	0.892 ± 0.011	0.815 ± 0.014	31.0	18.4
U-Net++	0.905 ± 0.009	0.832 ± 0.012	45.1	26.7
DeepLabV3+ (ResNet50)	0.911 ± 0.008	0.841 ± 0.010	54.7	31.2
CutisNet (proposed)	0.927 ± 0.007	0.862 ± 0.009	45.8	28.6

**Table 3:** Summary of the dimensionality of the extracted deep and handcrafted features, the total fused feature vector before feature optimization, and the final optimized feature vector obtained using Gray Wolf Optimization (GWO).

Features	Feature Vector Dimension
MobileNet	1280
DINO (ViT-S/8)	384
Tamura	6
Gabor	24
GLRLM	5
Symmetry	2
Ellipse Fitting	5
Hu Moments	7
Superpixel + Contour + Center	1280 (640 keypoints Ã—2D)
SIFT	1280 (e.g., 10 keypoints Ã—128)
Total Before Fusion	~5313
After GWO Feature Fusion	1856

**Table 4:** Comparison of final accuracies using different optimizers simultaneously.

**Optimizers**	GWO	GA	PSO	ACO	DE	SSA
**Accuracies %**	88.00	83.90	84.70	77.70	78.70	86.10

**Table 5:** Summary of the implementation details, training hyperparameters, Gray Wolf Optimization configuration, and experimental environment used in the proposed framework.

Category	Details
Deep Features	MobileNetV2: 1280 dims, DINO (ViT-S/8): 384 dims
Handcrafted Features	Tamura: 6, Gabor: 24, GLRLM: 5, symmetry: 2, ellipse fitting: 5, Hu moments: 7, superpixel + contour + center: 1280 (640 keypoints × 2D), SIFT: 1280
Total Fused Dimension	5313
After GWO	1856
Optimizer	Adam
Initial Learning Rate	0.001
Learning Rate Scheduler	ReduceLROnPlateau (factor = 0.1, patience = 3)
Batch Size	32 (classification), 16 (segmentation)
Max Epochs	100 with early stopping (patience = 10)
Cross-Validation	5-fold stratified (80/20 train–validation split per fold)
Gray Wolf Optimization	Population size = 30, max iterations = 50, control parameter linearly decreased from 2 to 0
Hardware	Google Colab Pro (NVIDIA Tesla T4 GPU, 16 GB VRAM)
Software Environment	Python 3.10, PyTorch 2.0.1, CUDA 11.8

**Table 6:** Computational cost analysis of the proposed framework, reporting the execution time and computational complexity (FLOPs) of each major processing and feature extraction module.

Module	Total Time (per Image)	MFLOPS Taken
Preprocessing	0.032194	92.03
Hair Removal	0.1951648	616.04
CutisNet Segmentation	0.141218	4.92
MobileNetV2	0.396477	300
ViT-Dino	0.983	21,000
Superpixel Centroid + Contour	0.565464	288.44
SIFT	0.1217	398.83
Tamura Descriptors	0.029072	12.8451
Gabor Texture	0.811491 s	311.9514
GLRLM	0.741	1344.09
Symmetry Analysis	0.023911 s	6.82
Ellipse Fitting	0.005171	2.0002
Hu Moments	0.000241	7.8643

**Table 7:** Classification metrics of the proposed model on various datasets.

Dataset	Accuracy	Precision	Recall	F1-Score	Sensitivity	Specificity	Balance Accuracy
PAD-UFES-20	0.880 ± 0.016 (0.864–0.896)	0.845 ± 0.021 (0.824–0.866)	0.840 ± 0.019 (0.821–0.859)	0.841 ± 0.020 (0.821–0.861)	0.840 ± 0.019 (0.821–0.859)	0.968 ± 0.008 (0.960–0.976)	0.900 ± 0.014 (0.886–0.914)
ISIC	0.945 ± 0.011 (0.934–0.956)	0.907 ± 0.014 (0.893–0.921)	0.910 ± 0.012 (0.898–0.922)	0.911 ± 0.013 (0.898–0.924)	0.911 ± 0.012 (0.899–0.923)	0.987 ± 0.006 (0.981–0.993)	0.949 ± 0.009 (0.940–0.958)
Derm7pt	0.836 ± 0.019 (0.817–0.855)	0.792 ± 0.025 (0.767–0.817)	0.790 ± 0.023 (0.767–0.813)	0.792 ± 0.024 (0.768–0.816)	0.792 ± 0.023 (0.769–0.815)	0.948 ± 0.010 (0.938–0.958)	0.870 ± 0.017 (0.853–0.887)
BCN20000	0.932 ± 0.013 (0.919–0.945)	0.877 ± 0.017 (0.860–0.894)	0.885 ± 0.015 (0.870–0.900)	0.880 ± 0.016 (0.864–0.896)	0.885 ± 0.015 (0.870–0.900)	0.984 ± 0.007 (0.977–0.991)	0.935 ± 0.011 (0.924–0.946)
PH^2^	0.967 ± 0.009 (0.958–0.976)	0.943 ± 0.011 (0.932–0.954)	0.950 ± 0.010 (0.940–0.960)	0.947 ± 0.010 (0.937–0.957)	0.947 ± 0.010 (0.937–0.957)	0.973 ± 0.007 (0.966–0.980)	0.962 ± 0.008 (0.954–0.970)

**Table 8:** Ablation analysis of each module on various datasets.

Module		Description	Accuracies (%)				
			PAD-UFES-20	ISIC	Derm7pt	BCN20000	PH^2^
Full Model		Model trained using all feature extraction techniques, including preprocessing, deep features extraction, lesion keypoints, textural descriptors, shape features	88.0	94.5	83.6	93.2	96.7
Without Preprocessing		Model trained without image preprocessing (resizing, color & illumination correction, contrast enhancement, smoothing)	82.00	86.90	76.90	88.10	89.20
Without Hair Removal		Model trained without Frangi and Gabor hair removal and Telea inpainting	84.20	89.50	80.10	91.20	92.30
Without Deep Features	MobileNet V2	Model trained without deep features from MobileNetV2	72.50	79.2	66.7	75.50	80.10
	ViT-Based DINO	Model trained without ViT-based DINO deep features	69.70	73.9	69.2	71.40	77.20
	MobileNet+ Dino	Model trained without deep features from MobileNetV2 and ViT-based DINO	61.90	67.50	66.00	61.80	71.40
Without Lesion Keypoints	SIFT + Superpixel Contour and Centroid	Model trained without SIFT keypoints and superpixel contour and centroid points	79.20	84.20	77.30	80.70	87.50
	SIFT	Model trained without SIFT keypoints	85.30	90.30	81.9	85.90	93.10
	Superpixel Contour and Centroid	Model trained without superpixel contour and centroid points	82.90	87.30	79.1	85.70	90.4
Without Textural Features	Gabor response + Tamura + GLRLM	Model trained without Gabor response, Tamura descriptors, and GLRLM textures	81.40	87.30	75.90	81.5	88.9
	Gabor Response	Model trained without texture features from Gabor response	85.30	92.10	80.90	85.70	90.50
	Tamura Descriptors	Model trained without texture features from Tamura descriptors	83.50	90.50	78.80	88.40	92.70
	GLRLM	Model trained without GLRLM textures	85.60	92.30	78.50	89.40	92.60
Without Shape-Based Features	Symmetry Analysis + Ellipse Fitting + Hu Moments	Model trained without symmetry analysis, ellipse fitting, and Hu moments	78.80	82.10	72.4	83.5	91.20
	Symmetry Analysis and Ellipse Fitting	Model trained without symmetry analysis and ellipse fitting	81.20	87.70	77.40	88.10	93.80
	Hu Moments	Model trained without Hu moments	86.40	90.20	80.20	91.40	94.80
Gray Wolf Optimizer		Model trained without GWO	71.90	78.40	68.90	78.60	83.70

**Table 9:** Comparative performance analysis across benchmark datasets.

Dataset	Author	Accuracy
PAD-UFES-20	Khurshid et al. [20]	0.851
	Pham et al. [21]	0.8435
	Khurshid et al. [22]	0.726
	Tang et al. [23]	0.830
	Uliana and Krohling [24]	0.6457
	Proposed model	88.0
ISIC	Moodi et al. [25]	0.7142
	Aboulmira et al. [26]	92.2%
	Gamil et al. [27]	91.00%
	Ozdemir et al. [28]	93.60%
	Yang et al. [29]	92.791%
	Pacal et al. [30]	0.9254
	Proposed Model	94.5
Derm7pt	Xu et al. [31]	0.813
	Bi et al. [32]	0.699
	Dosovitskiy et al. [10]	0.777
	Tang et al. [33]	0.785
	Proposed Model	83.6
BCN20000	Agarwal et al. [34]	91.17%
	Sharafudeen et al. [35]	87.33%
	Joshi et al. [36]	92%
	Gunaratne et al. [37]	91.9
	Proposed Model	93.2
PH^2^	Moodi et al. [25]	0.905
	Srivastava et al. [38]	0.91
	Nawaz et al. [39]	95.6%
	Toprak et al. [40]	94.44%
	Proposed Model	96.7

## Data Availability

The data presented in this study are available in the PAD-UFES-20 dataset is available at https://data.mendeley.com/datasets/zr7vgbcyr2/1c (accessed on 7 July 2026), the ISIC dataset is available at https://www.isic-archive.com/ (accessed on 7 July 2026), the Derm7pt dataset is available at https://derm.cs.sfu.ca/Download.html (accessed on 7 July 2026), the BCN20000 dataset is available through the https://challenge.isic-archive.com/data/#2019 (accessed on 7 July 2026), and the PH^2^ dataset is available at https://www.fc.up.pt/addi/ph2%20database.html (accessed on 7 July 2026).
